# Physician-modified aortic cuff to “stabilize” paravisceral aortic thrombus and minimize risk of distal embolization during pararenal aneurysm repair with fenestrated stent graft

**DOI:** 10.1016/j.jvscit.2022.05.002

**Published:** 2022-06-23

**Authors:** Brandon Douglass, Tiziano Tallarita, Jason Beckermann, Vinay Nijhawan, Joseph Wildenberg, Jeremy McBride, Thomas Carmody

**Affiliations:** a Surgery Department, Mayo Clinic, Rochester, MN; b Cardiovascular Surgery Department, Mayo Health System, Eau Claire, WI; c Surgery Department, Mayo Health System, Eau Claire, WI; d Radiology Department, Mayo Health System, Eau Claire, WI

**Keywords:** Physician modified graft, Complex aneurysm, Paravisceral aortic thrombus

## Abstract

As technology and surgeon experience evolve, endovascular repair of complex abdominal aortic aneurysms is often preferred in appropriately selected patients. However, the presence of pedunculated aortic thrombus represents a relative contraindication for endoluminal therapy due embolization risks. Here, we present a 68-year-old woman with a 5.8-cm pararenal aortic aneurysm associated with pedunculated aortic thrombus. She was treated with a modified Cook-Zenith aortic cuff to first entrap the aortic thrombus, followed by treatment of the aneurysm with a modified Z-FEN graft. This cuff modification provides a novel approach to deal with such luminal thrombus.

Endovascular repair of infrarenal abdominal aortic aneurysms has become the mainstay of treatment in patients with suitable anatomy.^1^, ^2^, ^3^ There are, however, anatomical and clinical features that make the use of endovascular treatment challenging or impossible.^4^, ^5^ These features include early renal artery bifurcation, extensive and pedunculated aortic thrombus (shaggy aorta), inadequate iliac access, advanced chronic kidney disease, connective tissue disorders. and younger age, to name a few. Pedunculated intraluminal thrombus is one risk factor that increases the morbidity of endovascular repair. A staging system to characterize thrombus and assess risk has been described by Oderich et al.^5^ Based on this staging system, the risk of visceral embolization increases according to the extent and intraluminal projection of the aortic thrombus. Here, we discuss a patient with a single functional kidney threatened by pedunculated thrombus in a large pararenal aortic aneurysm. The patient refused open surgical repair and underwent a novel endovascular approach to treat her aneurysm and entrap the aortic thrombus without jailing the target vessels, mitigating the risk of visceral embolization. The patient gave their consent for publication.

## Case report

**Fig 1:**
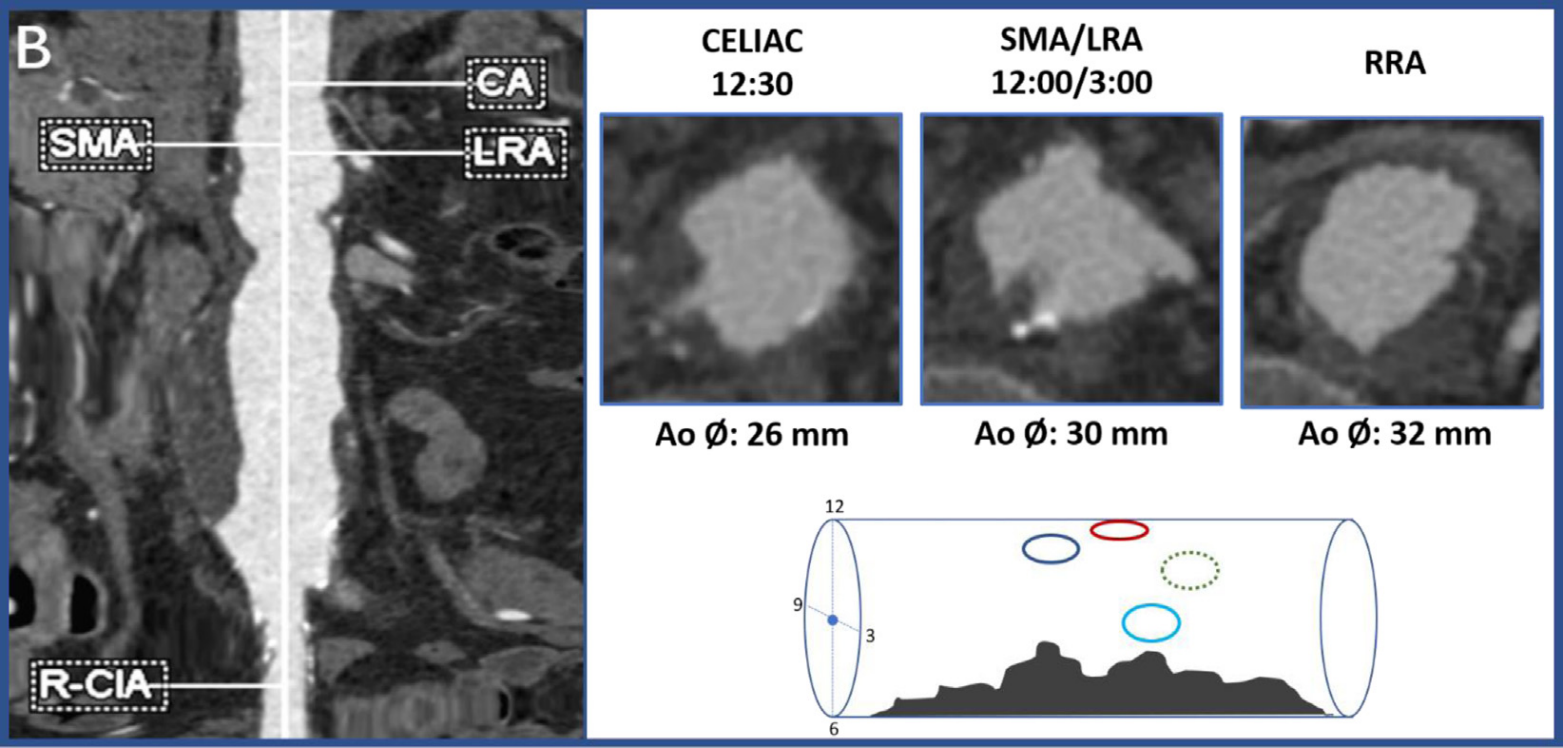
(Right) Center line measurement of the abdominal aorta with extension into the right common iliac artery (corresponding visceral arterial levels labeled). The aneurysm starts at the level of the renal arteries. The maximal diameter of the aneurysm is 58 mm. (Left) Computed tomography scan axial cuts of the aorta at the level of celiac artery (dark blue circle), superior mesenteric artery/LRA (red circle and ∗light blue circle respectively), and right renal artery (∗∗dotted green circle). Note: Schematic on bottom is oriented superior on left, inferior on right and the patient’s right projecting toward the reader. ∗Light blue circle projects out toward the reader. ∗∗Dotted green circle projects away from the reader. *LRA*, left renal artery; *RRA*, right renal artery; *SMA*, superior mesenteric artery.

A 68-year-old woman with hypertension, hyperlipidemia, obesity (body mass index of 34), a 50-pack-year smoking history, and a recent diagnosis of malignant hypertension was referred to our clinic after a computed tomography angiogram had shown a 5.8 cm pararenal aortic aneurysm (Fig 1). She also had a long segment, near occlusion, of the right renal artery with associated atrophic right kidney. The mesenteric arteries and left renal artery (LRA) were widely patent. Interestingly, there was a pedunculated thrombus in the mesorenal segment of the aorta, starting at the level of the celiac trunk and extending to the LRA (Fig 1). The thrombus involved approximately 180° of the posterior aortic lumen, with finger-like projections between 3 and 11 mm in depth (Fig 1). At the time of our initial consultation, her hypertension had been well-controlled with three antihypertensive medications and her creatinine was 1.2 mg/dL. Both common iliac arteries had aneurysmal changes, measuring 18 mm (right) and 20 mm (left) in maximum diameter distally. The patient had no prior cross-sectional abdominal imaging; therefore, the rate of growth was unknown.

### Backtable surgery

**Fig 2:**
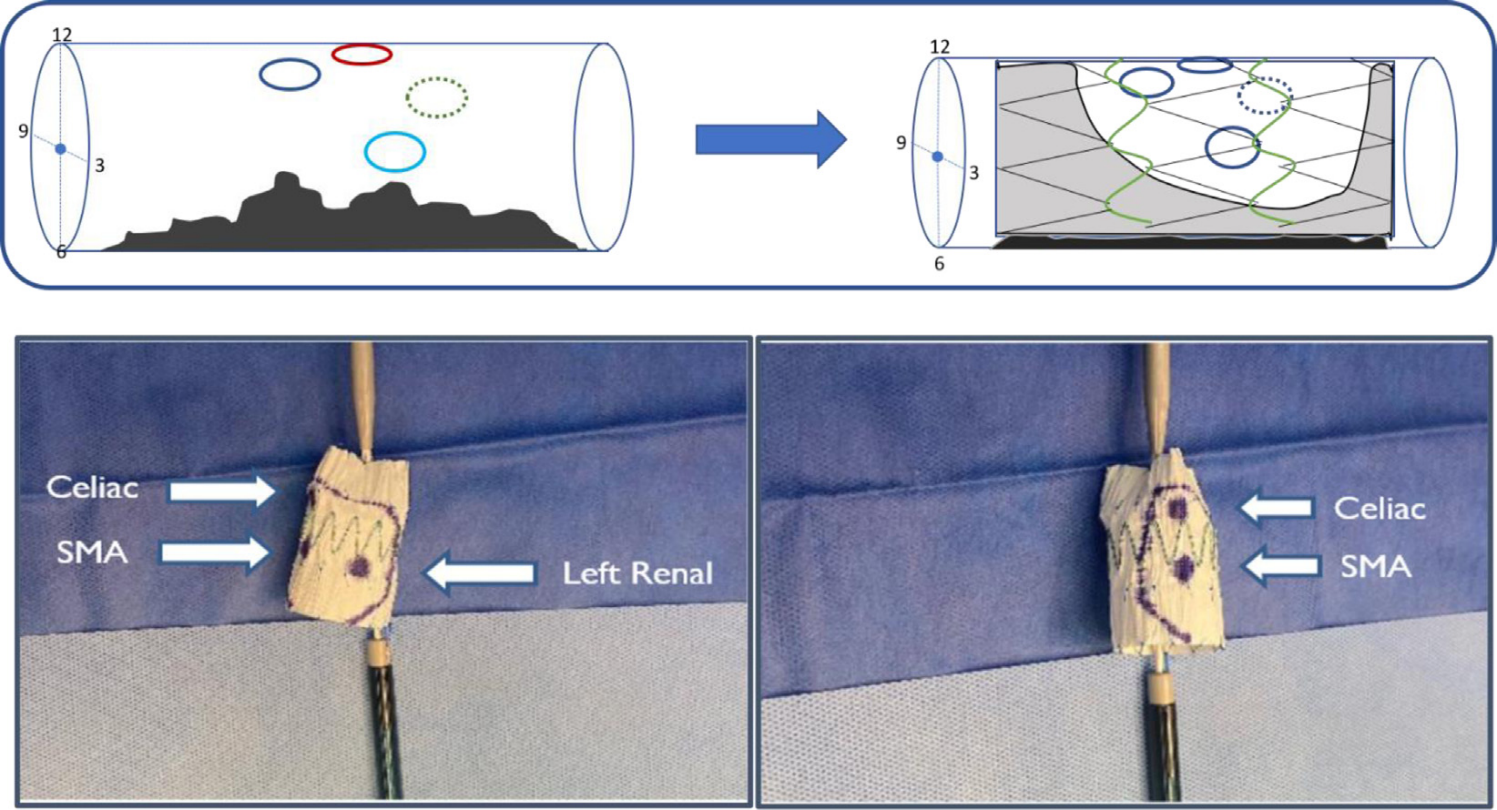
(Top) Proposed locations of relevant visceral vessels and the corresponding proposed landing of the first stage graft with large anterior fenestration and intact posterior fabric. (Bottom) The 32 mm × 58 mm aortic cuff in the process of being modified. Arrows depict the locations of the celiac artery, superior mesenteric artery (*SMA*), and left renal artery (LRA) corresponding with their relative locations on the patient. The outline depicts the proposed area of fabric removal, approximately 40% of the circumference.

We elected to proceed with an endovascular repair of the aneurysm. A Cook Zenith aortic cuff (Cook Medical, Bloomington, IN) 32 mm × 58 mm was modified (Fig 2). First the celiac, SMA, and LRA positions were accurately marked on the graft according to the clock position and central line flow measurements.

**Fig 3:**
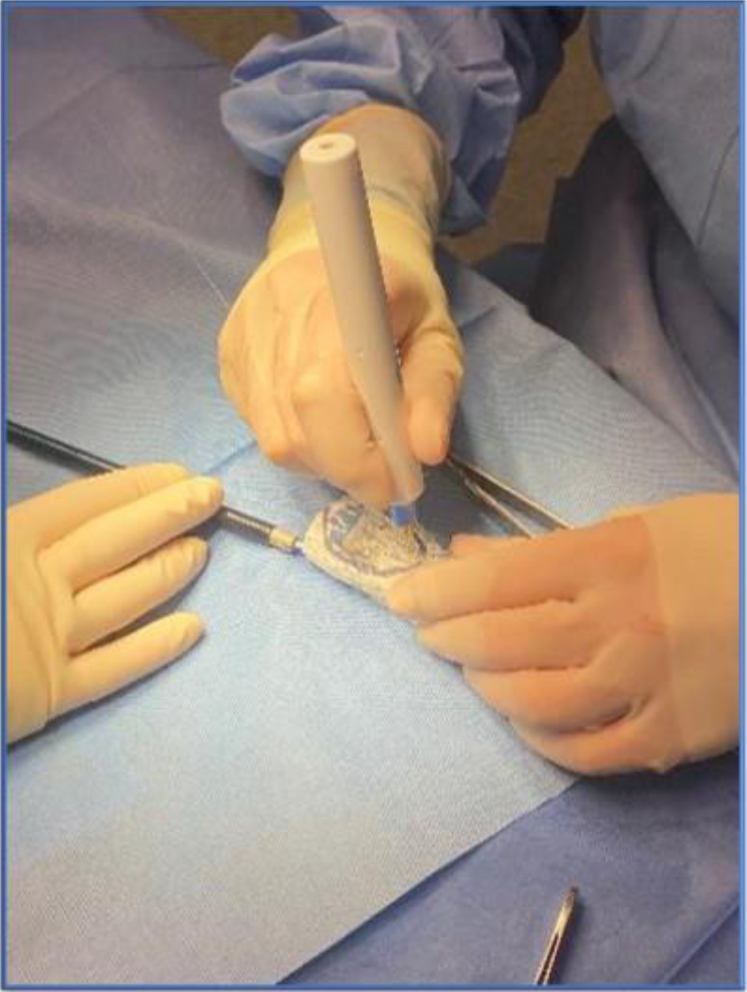
Removing fabric from anterior 40% of the first stage 30 × 58 mm stent graft using a fine tip high temperature cautery device.

The celiac artery, SMA, and LRAs were at 12:30, 12:00, and 3:00, respectively (Figs 1 and 2), occupying approximately 30% of the aortic circumference. Based on these data, 40% of the fabric of the stent graft was removed from the anterior aspect of the graft using a high temperature fine tip cautery device (Figs 2 and 3). The section of fabric removed corresponded with the location of the celiac, SMA, and LRA, and this section was extended outward 0.5 cm each side to account for possible measurement errors and stent bulging (Figs 2 and 3).

**Fig 4:**
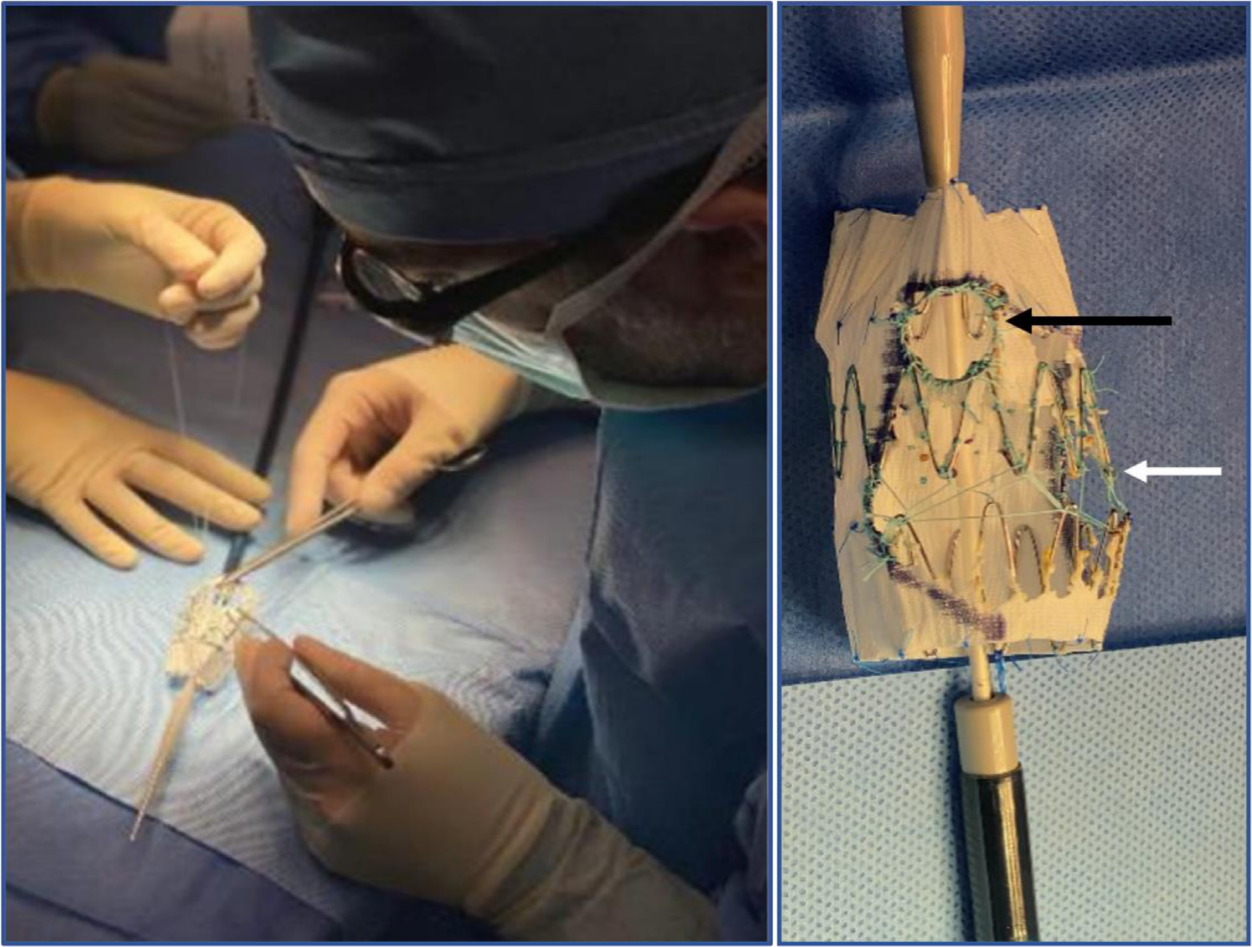
(Left) Suturing of the free struts using nonabsorbable suture for stability. (Right) Final result after all modifications: 40% of the fabric removed anteriorly, free struts interconnected by Ethibond suture (white arrow), and creation of the virtual fenestration corresponding to the celiac artery (black arrow). The virtual fenestration is sutured to the struts without involving fabric.

The right renal artery was not incorporated in the stent graft because it was near occluded from the origin to the renal hilum, making its revascularization not technically feasible. In addition, the right kidney was considered not salvageable because of its maximal diameter of only 8 cm. Next, the bare metal struts, being free of fabric and bulging out were stabilized and interconnected using a nonabsorbable 5-0 Ethibond suture (Fig 4). This idea was inspired by the Cook dissection stent, where the apex of each strut is connected with Ethibond suture in a zigzag fashion to minimize stent bulging and the risk for incorrect deployment. For the purpose of accurate and efficient orientation of the cuff, a 15 mm × 15 mm virtual fenestration was created corresponding with the location of the celiac artery, using a radiopaque wire sutured around the struts without any fabric involved (Fig 4).

**Fig 5:**
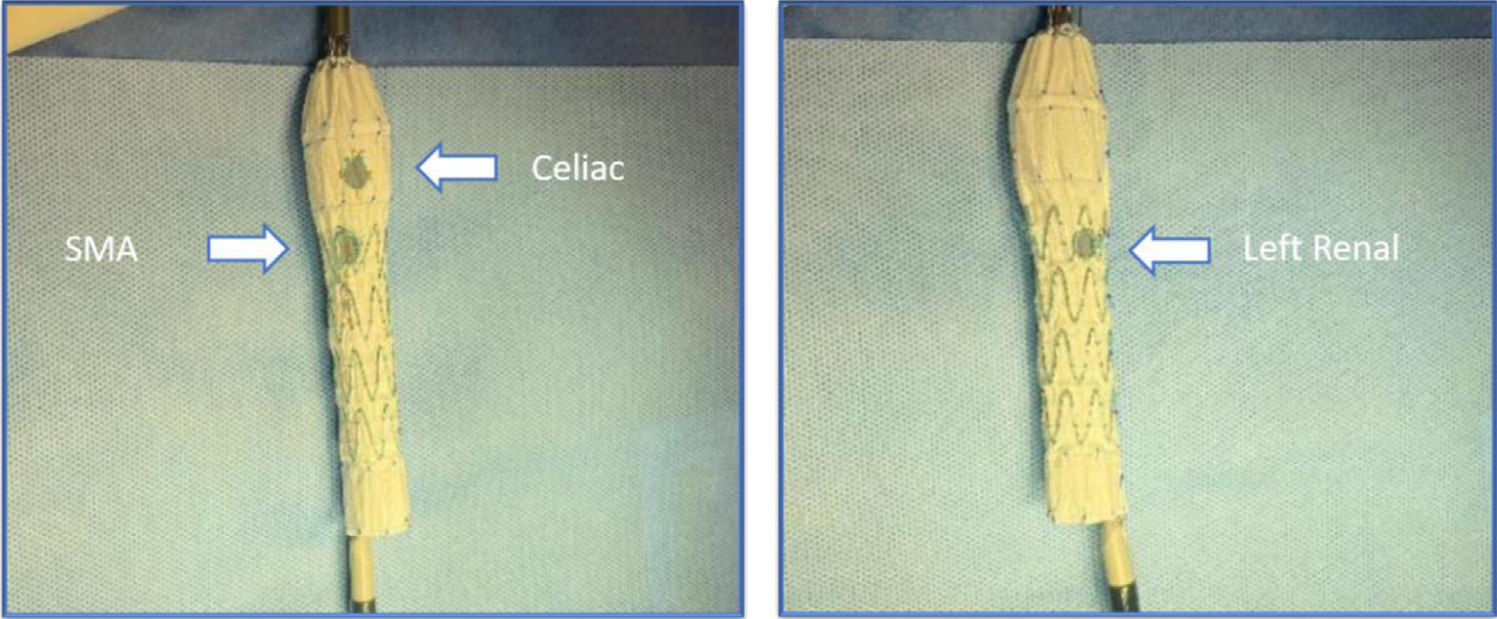
Second stage 32-mm fenestrated graft showing the locations of the celiac artery, superior mesenteric artery (*SMA*), and left renal artery (LRA) fenestrations. The LRA was modification was added by the surgeon.

The virtual fenestration was preloaded with an 0.018-inch wire. A constraining wire was run on the back of the stent graft to obtain a decrease in the diameter of approximately 40% of the stent, thereby minimizing friction between the stent and the thrombus during the stent manipulation. Now constrained, the modified aortic cuff was re-sheathed. The second part of the back-table modification was the addition of an extra fenestration to the Z-FEN stent graft. The added fenestration allowed placement of the stent graft in the supraceliac region (Fig 5). Following modification, the Z-FEN stent graft was resheathed. Criteria provided by the manufacturer state that Z-FEN stent grafts are made to treat juxta renal aortic aneurysms, in general landing at the level of the SMA. Therefore, in this case, an off-the-shelf Z-FEN would have landed in the middle of the aortic thrombus, likely resulting in thrombus disruption and distal embolization.

### Procedure

**Fig 6:**
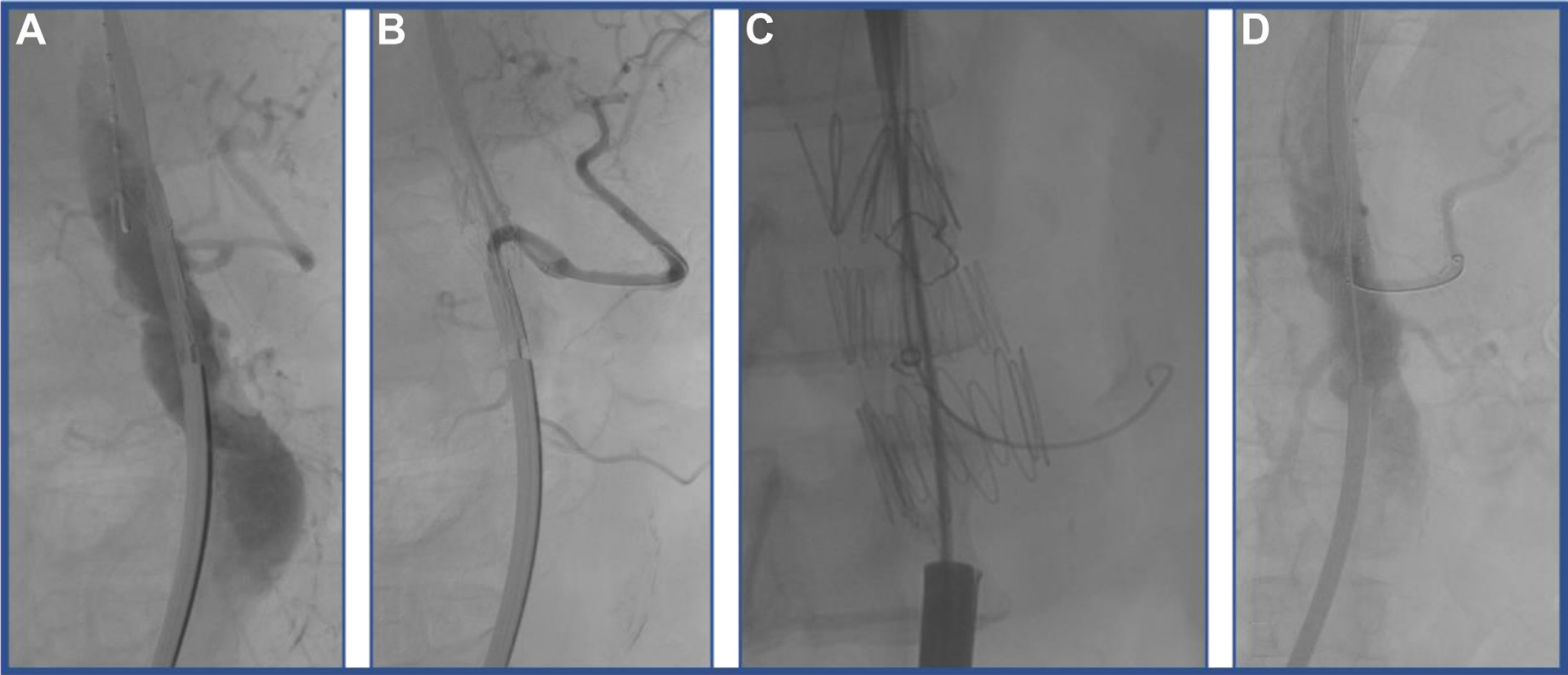
**(A)** Alignment of the virtual fenestration of the aortic cuff with the celiac trunk. **(B)** Partial deployment of the cuff, which opens the fenestration for cannulation. The celiac artery is cannulated through the fenestration with a Kumpe catheter and soft glidewire while maintaining the graft 40% constrained. **(C)** The glidewire is exchanged to a Rosen wire and a 7F sheath is advanced inside the celiac trunk to stabilize the graft; the graft is then completed deployed by releasing the constraining wire. **(D)** Completion angiogram showing widely patent celiac, superior mesenteric artery (SMA), and left renal artery (LRA).

Toward the end of the Z-FEN graft modification, the patient was brought to the operative room and placed on supine position. Bilateral percutaneous access of the common femoral artery was obtained. Both accessed arteries were preclosed with Perclose devices (Abbott Vascular, Santa Clara, CA), two on each side, followed by placement of two 8F sheaths. Surgical access of the left brachial artery was also obtained, and a 12F Gore DrySeal (W. L. Gore & Associates, Flagstaff, AZ)sheath placed. A heparin bolus was given with a goal activation clotting time of more than 250 seconds. Each access site was obtained while carefully avoiding crossing the aortic thrombus. The modified aortic cuff was oriented extracorporeally with the virtual fenestration at 12:00 o’clock and the right groin access was upsized to a 20F Cook sheath. The portion of the aorta with the thrombus was then crossed with a soft glidewire and the wire was snared from the brachial sheath. By using a long tibial sheath, extending from the brachial artery sheath to the right femoral sheath, both the constraining wire and preloaded virtual fenestration wire were exteriorized through the brachial access. An angiogram was performed from above, identifying the celiac trunk origin. The cuff was introduced through the right femoral 20F sheath with the tip positioned in proximity to the celiac trunk, with the virtual fenestration aligned (Fig 6, *A*). The cuff was partially unsheathed to expose the virtual fenestration. The virtual fenestration and the celiac trunk were cannulated from above with a Kumpe catheter and glidewire, subsequently exchanged to a Rosen wire (Figs 6, *B*). A 7F Raabe sheath was then advanced through the fenestration and into the ostium of the celiac trunk to stabilize the graft during its deployment. Importantly, the graft was still 40% constrained to minimize friction against the thrombus and to maintain the ability to fine tune placement and orientation. With the 7F sheath still canulating the celiac trunk, the constraining wire was removed, and the stent was fully deployed (Fig 6, *C*). The stent was not post-ballooned to avoid “tooth-pasting” of the thrombus. A completion angiogram confirmed continued patency of the celiac artery, SMA, and LRA (Fig 6, *D*). With the thrombus entrapped, we could proceed with the deployment of the modified fenestrated stent graft.

**Fig 7:**
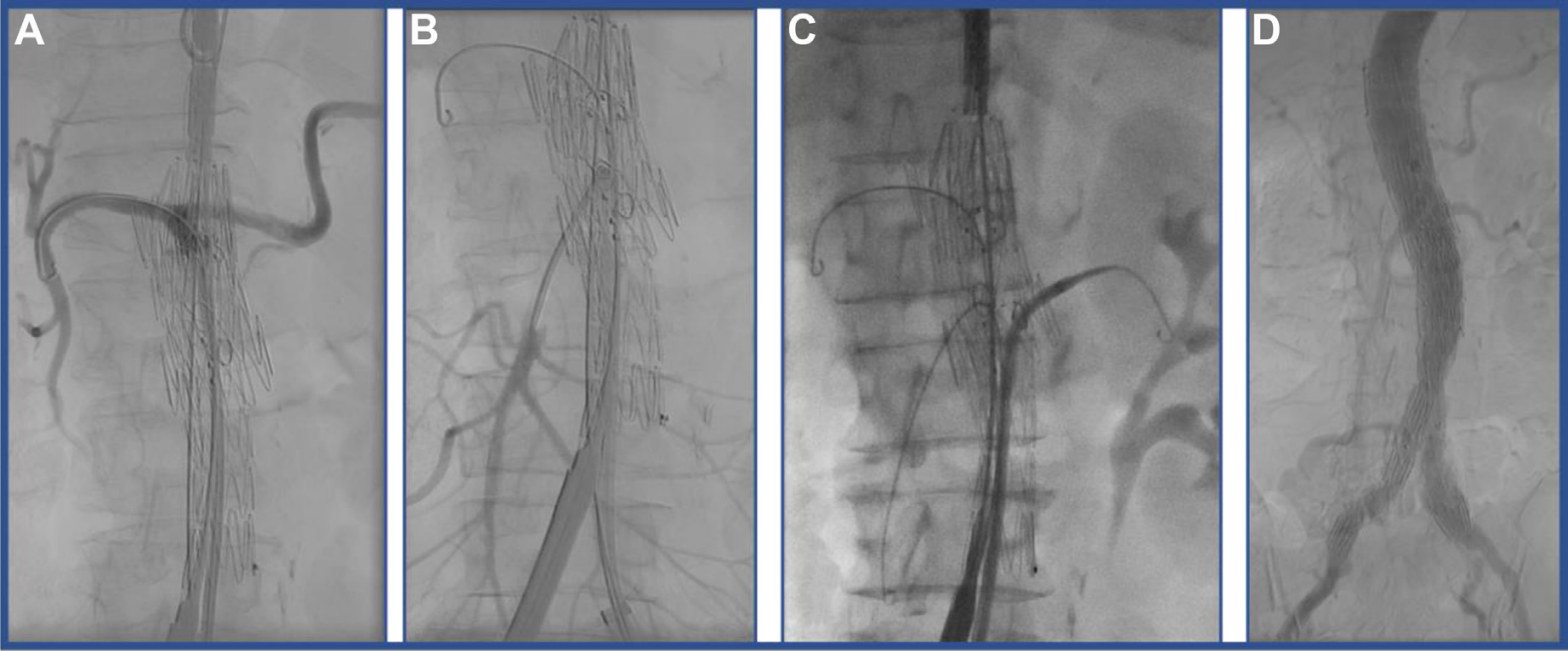
Partial deployment of the modified Z-FEN stent graft, with sequential cannulation of the celiac artery **(A)**, superior mesenteric artery (SMA) **(B)**, and left renal artery (LRA) **(C)**. After deployment of the bifurcated component, all the attachments were ballooned. Completion angiogram showed widely patent stents, no type I or III endoleak, contrast extravasation, or dissection **(D)**.

The left femoral access was upsized to a 16F Cook sheath. The modified Z-FEN was oriented extracorporeally and introduced from the right femoral access. The graft was fully unsheathed but still constrained to allow stent manipulation (Fig 7, *A*). Each fenestration was cannulated, sequentially in a cranial to caudal fashion from the left groin with a Kumpe catheter and the adjunct of a steerable sheath. For all three target vessels, the glidewire was exchanged for a Rosen wire (Fig 7, *B* and C). The 5F sheaths were advanced into the LRA and SMA to stabilize the Z-FEN in preparation for its full deployment. The constraining mechanism was then released and the stent graft fully deployed. The stent graft was ballooned to improve apposition to the aortic wall. Each target vessel was stented with Gore VBX stent graft sequentially, in a cranial to caudal fashion. Each target stent was flared inside the main aortic body with a 10 mm × 20 mm balloon. With the visceral artery stents in place, the bifurcated component of the stent graft was deployed, landing the iliac limb in the distal common iliac artery, bilaterally. Stent attachments were ballooned to achieve adequate wall apposition. Completion angiogram showed no type I or III endoleak, dissection, or contrast extravasation, with all three target vessels being widely patent (Fig 7, *D*).

**Fig 8:**
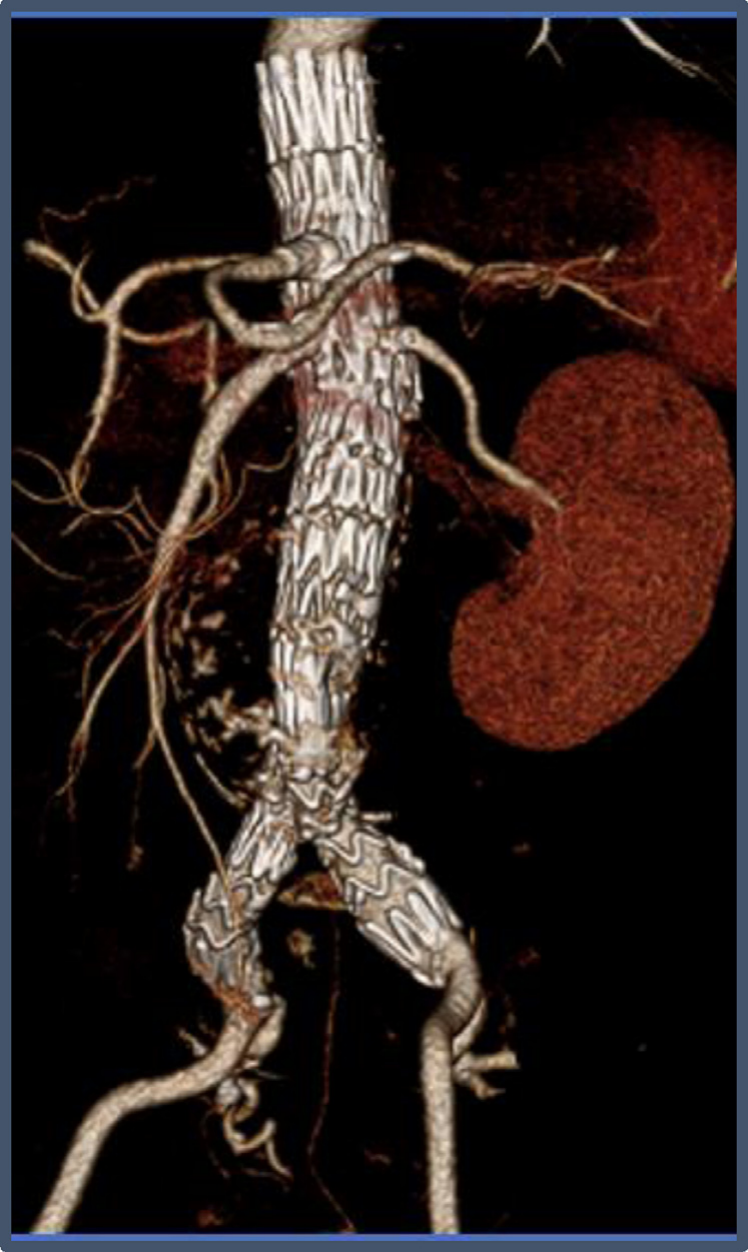
Three-dimensional reconstruction of a postoperative computed tomography scan performed in follow-up of the final graft in situ.

The patient had an uneventful hospital stay and was discharged home on postoperative day 4 (a -day 1 in intensive care). Follow-up imaging at 6 weeks and 3 months showed widely patent visceral stents and no type I or III endoleak (Fig 8). A left renal ultrasound examination confirmed a widely patent left renal stent, and her renal function was at her preoperative baseline with a creatinine of 1.2 mg/dL.

## Discussion

We present a novel technique where a modified Cook aortic cuff with anterior fabric removal allowed preservation of flow to the meso-renal vessels. The intact posterior fabric of the cuff fully entrapped the posterior para-visceral aortic thrombus, minimizing distal embolization risk. The virtual fenestration of the cuff was oriented extracorporeally to decrease cuff manipulation during its delivery and was placed in approximately 15 minutes. Complex aortic aneurysm repair with fenestrated stent grafts can require several hours of stent manipulation to align the fenestrations to the target vessels and, in presence of pedunculated aortic thrombus, it may result in thrombus disruption and distal embolization. The first stage with thrombus entrapment by the aortic cuff was swiftly done to allow safe alignment of the modified Z-FEN.

**Fig 9:**
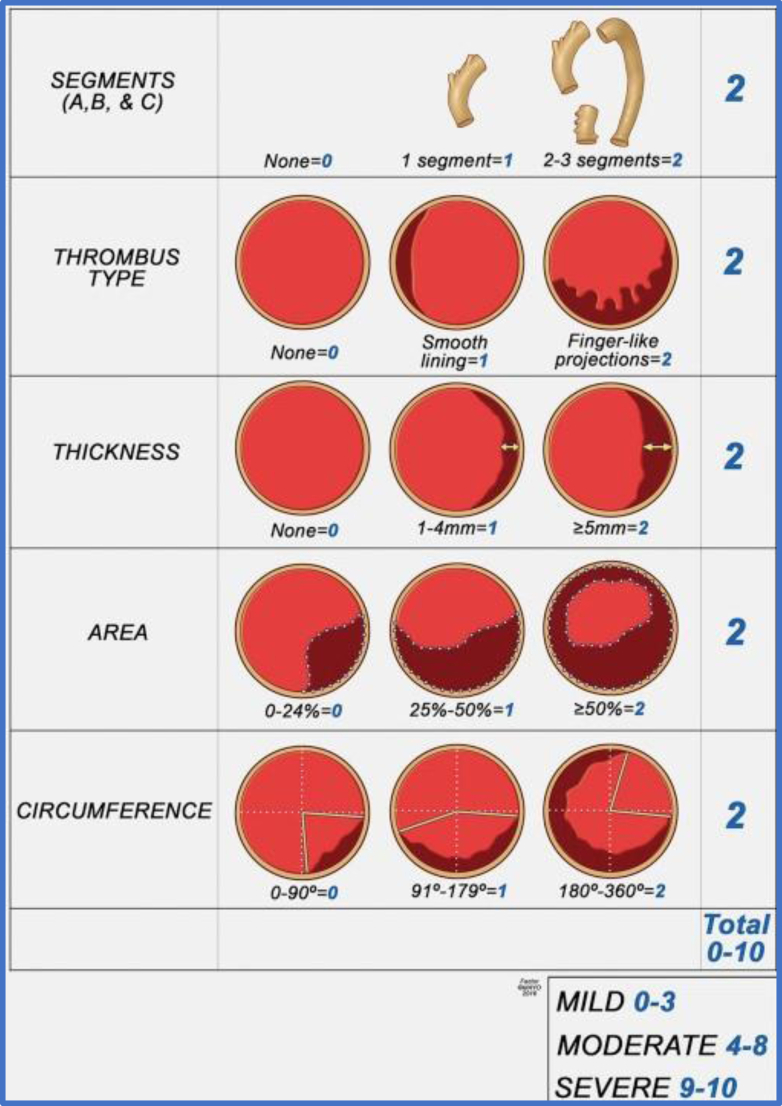
Qualitative score-based classification of aortic wall thrombus (AWT) proposed by Oderich et al. Classification severity: Mild AWT ranges from 0 to 4, moderate AWT from 4 to 8, and severe AWT scores 9 and 10 with each individual item graded from 0 to 2.^5^

Approximately 70% to 80% of patients with AAAs have associated intraluminal thrombus adherent to the vessel wall.^5^ The high prevalence of thrombus within the vessel wall can increase the operative and postoperative morbidity and mortality, particularly when high risk features are present.^5^^,^^6^ These high-risk features include multiple involved segments, finger-like projections, a thrombus thickness more than 5 mm, thrombus occupying greater than 50% of the lumen, and thrombus occupying greater than 180° of the circumference.^5^ When a patient meets the criteria for AAA repair and has an accompanying intraluminal thrombus with high-risk features, safe endovascular repair can be challenging. The risks of distal embolization and associated visceral organ injury are important to consider when deciding between an open surgical versus endovascular approach. Based on Oderich’s staging of intraluminal aortic thrombus,^5^ our patient had moderate- to high-risk thrombus features with the highest risk for visceral embolization (Fig 9). Despite the patient being fit for an open repair, she declined. Therefore, an endovascular repair was pursued with the aim of stabilizing the thrombus first. The purpose of the modified cuff was not to provide structural integrity to the abdominal aortic aneurysm repair or to seal off the aneurysm, but rather to entrap the thrombus and thereby decrease the risk of embolization. Once the modified Z-FEN stent graft was in place, the cuff became endotrash.

There were several challenges associated with the modification of this aortic cuff that we needed to address. First was the accuracy in removing enough fabric to spare the origins of all target vessels, while leaving enough fabric to cover and entrap the entire paravisceral aortic thrombus. We opted to remove approximately 40% of the fabric, knowing that the visceral arteries occupied approximately 30% of the circumference of the vessel, which allowed some margin of error in alignment. Second, we aligned the bare metal part of the stent graft precisely, while avoiding accidental coverage of one or more visceral arteries, which would result in a poor outcome. This was accomplished by creating a large, 15 mm × 15mm, virtual fenestration for the celiac artery with a radiopaque marker. The celiac artery was, in fact, the center of the uncovered portion of the stent, allowing for easy alignment. The fenestration was precannulated with an 0.018-inch wire for easier access, to minimize intraluminal manipulation, and decrease thrombus friction. By removing the fabric, the unconstrained bare metal stents were going to bulge out. To stabilize the free struts, we took inspiration from commercially available Cook dissection stent and interconnected the struts with a nonabsorbable, 5-0 Ethibond suture (Fig 5).

Currently, paravisceral aneurysm can be treated by manufactured fenestrated stent graft within a trial only (Z-FEN plus from Cook, TAMBE from Gore or off the shelf fenestrated-branch stent grafts). Outside the trial, the commercially available Z-FEN stent graft is designed to treat short neck infrarenal/juxtarenal aneurysms. Other options include physician modified graft or parallel grafts. This patient had a pararenal aortic aneurysm; therefore, a Z-FEN was not an option. We opted to modify a Z-FEN stent graft by adding an extra fenestration for the LRA to obtain at least 2 cm of healthy, proximal aortic neck. The advantages of doing so, compared with modifying a thoracic stent graft, were (a) a much shorter modification time, (b) a decrease in how many times the fabric was pierced, and (c) the cost (approximately $8000 less in costs). A physician-modified thoracic stent graft would have required to pierce the fabric numerous times (in this case it would have been three fenestrations, constrain wire, six to eight reducing stitches on the back of the stent, and all the markers), increasing the risk of endoleak or damage of the stent graft itself. The drawbacks of modifying a Z-FEN were the need to wait 3 to 5 weeks for the graft (therefore, it could not be used for an urgent case), the larger profile of the device, and inability to treat a thoracoabdominal aneurysm.

## Conclusions

We proposed the consideration for a physician modified cuff technique, such as the one described here, in patients who are not candidates for open repair and but have moderate- to high-risk features for distal embolization during endovascular treatment. This novel stent graft modification allowed the authors to safely address a pedunculated luminal thrombus in the visceral segment of the aorta, while minimizing the risk of embolization during fenestrated stent graft repair. This novel technique of 40% bare metal/60% covered stent with virtual fenestration for orientation is a new addition to the endovascular armamentarium and could furnish an idea for future manufactured stent graft. Such a technique illustrates the importance of personalization and innovation with respect to patient care in endovascular surgery, especially in patients with complicated aneurysms who are not candidates or are unwilling to undergo an open repair.
